# Nephropathogenic infectious bronchitis virus-induced pyroptosis of chicken renal tubular epithelial cells via the MDA5/NF-κB/NLRP3 signalling pathway

**DOI:** 10.1186/s13567-025-01651-4

**Published:** 2025-12-01

**Authors:** Bingqing Zhang, Mengbing Ding, Yizhou Zeng, Jingyan Luo, Jiaqi Li, Xiaona Gao, Ping Liu, Gaofeng Cai, Zhanhong Zheng, Xiaoquan Guo

**Affiliations:** Jiangxi Provincial Key Laboratory for Animal Health, Institute of Animal Population Health, College of Animal Science and Technology, Jiangxi Agricultural University, Nanchang, 330045 China

**Keywords:** NIBV, pyroptosis, renal tubular epithelial cells, MDA5/NF-κB/NLRP3 signalling pathway

## Abstract

MDA5 is an innate pattern recognition receptor that is involved in the recognition of various viruses. It can recognize RNA viruses, activate downstream signalling pathways, facilitate the transcription of inflammatory factors, and induce cell pyroptosis. Pyroptosis is a form of programmed cell death accompanied by the release of inflammatory factors and an inflammatory response. In this study, we hypothesize that pyroptosis is elicited by the signalling cascade subsequent to the recognition of nephropathogenic infectious bronchitis virus (NIBV) by MDA5. Thus, we infected chicken renal tubular epithelial cells with NIBV and discovered that NIBV infection induced pyroptosis and increased the mRNA levels of MDA5. Consequently, we infected primary chicken renal tubular epithelial cells with NIBV and inhibited TRAF6 expression using the exogenous inhibitor C25-140. We found that NIBV could increase lactate dehydrogenase (LDH) levels, increase the proportion of pyroptotic cells, and increase the mRNA and protein levels of the MDA5/NF-κB signalling pathway and the classical pyroptosis pathway. Here, we selected the ubiquitin ligase TRAF6, a key node in the MDA5/NF-κB signalling pathway, from molecular biological and genetic perspectives to explore the molecular mechanism of NIBV-induced pyroptosis. After the inhibitor C25-140 was used, NIBV-induced apoptosis and the activity of the MDA5/NF-κB/NLRP3 pathway were reversed. In addition, the amount of NIBV replication in the cells was reduced. In conclusion, the MDA5/NF-κB/NLRP3 signalling pathway is involved in the regulation of pyroptosis in a NIBV-infected chicken renal tubular epithelial cell model. The inhibition of this signalling pathway can alleviate NIBV-induced pyroptosis and reduce the replication of NIBV in cells, which could become one strategy for treating NIBV.

## Introduction

Nephropathogenic infectious bronchitis is an acute infectious disease of poultry induced by nephropathogenic infectious bronchitis virus (NIBV). In kidneys infected with NIBV, ureteral pallor and urate deposition are accompanied by tubular patterns of proteins and cells and urate crystal deposition in renal tubules [1]. In terms of ultrastructure, the virus replicates in the renal tubules and collecting tubules, but it is more easily recruited to the collecting tubules and distal convoluted tubules, leading to vacuolation of the tubular epithelial cells, accompanied by obvious inflammatory cell infiltration [2].

Pyroptosis is a mode of cell death accompanied by an inflammatory response. Classical pyroptosis involves the recognition of pathogen-related molecular patterns and dangerous molecular patterns, the activation of downstream signalling pathways, the assembly of caspase-1 and ASC (apoptosis—associated speck—like protein containing a CARD), and inflammasome formation [3]. Cleavage of IL-1β and IL-18 induces cell membrane perforation and the release of inflammatory factors [4]. Coronavirus infection can activate inflammasomes and induce apoptosis, pyroptosis and necrotic apoptosis in specific cells [5]. NIBV-induced pyroptosis involves a variety of molecular processes [2]. However, the underlying mechanism of pyroptosis in NIBV remains unclear and needs to be further explored.

MDA5 is a pattern recognition receptor capable of recognizing RNA viruses, which subsequently stimulates innate immunity and inflammation and triggers a cascade of activation signals [6]. At the peak of viral infection, overactivation of the MDA5 receptor leads to dysregulation of a variety of complex signals and overactivation of the NF-κB inflammatory signalling pathway. After recognizing the virus, MDA5 undergoes a conformational change and interacts with the downstream adaptor protein IPS-1 located on the mitochondrial membrane [7] to recruit the key protein tumour necrosis factor (TNF) receptor-associated factor 6 (TRAF6) to induce TAK1-mediated IKK complex activation [8], which in turn activates the transcription factor NF-κB [9]. As a key immune mediator, NF-κB plays a key role in the activation of the NLRP3 inflammasome and can induce the release of IL-1β and IL-18 [10]. TRAF6 is ubiquitinated during the activation of the NF-κB signalling pathway and is an important regulator of NF-κB. However, the role of the MDA5/NF-κB/NLRP3 innate immune signalling pathway in NIBV-induced kidney inflammation is unclear.

In this study, renal tubular epithelial cells were extracted from 3–7-day-old chicks and infected with NIBV. We verified that NIBV induced kidney inflammation in chickens by activating pyroptosis through the MDA5/NF-κB/NLRP3 signalling pathway, providing new insights into the molecular regulatory mechanism of NIBV.

## Materials and methods

### Main materials and reagents

An LDH assay kit was purchased from Nanjing Jiancheng Bioengineering Institute (Jiangsu, China). A HOECHST/PI apoptosis detection kit and a YO-PRO-1/PI apoptosis detection kit were purchased from Beyotime (Jiangsu, China). DMEM and type I collagenase were purchased from Solarbio (Beijing, China). Foetal bovine serum was purchased from ExCell Bio (Jiangsu, China). A multifunctional spectrophotometer was produced by Bio-Rad Bio (California, USA). A Cell Counting Kit-8 was purchased from Yeasen Bio (Shanghai, China). All antibodies were purchased from Wanleibio (Shenyang, China). The gene primers used were synthesized by Qingke (Jiangsu, China). The TRAF6 inhibitor C25-140 was purchased from SELLECK Corporation (Houston, USA).

### Isolation and culture of primary renal tubular epithelial cells

Chicks aged 3–7 days were uniformly raised in a constant-temperature box, fed and watered together, and randomly chosen blindly, after which their kidneys were placed in a Petri dish containing 10% FBS and 2.5% DMEM. The kidney tissues were then extracted with tweezers and ground. The ground tissue was digested with type I collagenase. After digestion, a 200-mesh sieve was used for filtration. Finally, the renal tubular epithelial cells were suspended in DMEM and inoculated with NIBV at a concentration of 1 × 10^6^ cells/mL. When the growth density of the renal tubular epithelial cells reached 60–80%, the cells were infected with NIBV. The multiplicity of infection is 1 MOI. The detailed operation methods can be found in Liu et al. [11] and Li et al. [5].

### Lactate dehydrogenase (LDH) release assay

After infection with NIBV for 36 h, the culture medium of renal tubular epithelial cells was collected and centrifuged at 1200 rpm, after which the supernatant was aspirated. Lactic dehydrogenase activity was measured with a Lactate Dehydrogenase Content Detection Kit (Nanjing Jiancheng Bioengineering Institute, China). Using a multifunctional spectrophotometer (Bio-Rad, USA), the absorbance was measured at a wavelength of 450 nm, and the calculation was carried out according to the formula in the instruction manual. The formula for determining LDH activity (U/gprot) is as follows: (assay wells − control wells/standard wells − blank wells) × concentration of the standard solution of 0.2 μmol/mL/concentration of the sample protein in gprot/mL.

### Flow cytometry

The lower adherent primary renal tubular epithelial cells were digested with trypsin, and then the suspended cells in the supernatant were recovered, gently mixed, and centrifuged; the supernatant was discarded, and the bottom cells were collected. Afterwards, the cells were collected after the administration of 20 μM TRAF6 inhibitor. A YO-PRO-1/PI apoptosis and necrosis detection kit (Beyotime Bio Co., China) and flow cytometry were used to identify cells from the four time periods. The cell fluorescence intensity was analysed using FlowJo 10.0.0.

### Hoechst stains

For Hoechst/PI staining, we discarded the medium and gently washed the adhered primary renal tubular epithelial cells twice with PBS. The cells were treated with trypsin in a centrifuge tube and centrifuged at 500 rpm for 2 min. The cells were stained according to the Hoechst/PI staining kit (Beyotime Bio Co., China) instructions to detect pyroptosis. After half an hour, images were taken using a fluorescence microscope and analysed via the ImageJ program [12].

### Interference of NIBV-infected cells with the TRAF6 inhibitor C25-140

First, to detect the cytotoxicity of C25-140, we used 6 concentrations—0 M, 5 M, 10 M, 20 M, 30 M, and 50 M—and then used a Cell Counting Kit-8 (Yeasen Bio Co., Shanghai, China) to determine the cell viability at each concentration to determine the optimal concentration of C25-140.

To further study the mechanism through which signalling is involved in viral replication, we detected the mRNA and protein expression levels of the MDA5/NF-κB/NLRP3 signalling pathway. Renal tubular epithelial cells were divided into a control group, a control + C25-140 group, a NIBV group and a NIBV + C25-140 group. After the cells were infected with NIBV, the optimal concentration of C25-140 was added to the culture medium. The control group was cultured with conventional DMEM. After 36 h of continuous culture, the supernatant was discarded, and the cells were washed twice with PBS. TRIzol^®^ (Vazyme Bio Co., NanJin, China) was used to extract RNA for subsequent RT‒PCR experiments, and RIPA lysis buffer was used to extract protein for WB experiments.

### Quantitative real-time PCR

Total RNA was extracted from the sample using TRIzol^®^ (Vazyme Bio Co., NanJin, China) reagent according to the manufacturer’s protocol. After extraction, the OD260/OD280 of the RNA was between 1.8 and 2.2, indicating good quality. A total of 500 ng of total RNA from each sample was subsequently used for cDNA synthesis with a TransGen Biotech reverse transcription kit. cDNA was synthesized with a cDNA synthesis kit from TransGen Biotech Company (Beijing, China). After synthesis, the cDNA samples were stored at −20 °C. The MDA5/NF-κB innate immune pathway genes MDA5, IPS-1, TRAF6, TAK1, IKKα, IKKβ, NF-κB, P65, NLRP3, caspase-1, IL-1β, and IL-18 and the housekeeping gene GAPDH were obtained from the NCBI GenBank [13]. The upstream and downstream primer sequences of the target genes designed by BLAST at NCBI were sent to Shanghai Qingke Biological Company for synthesis. The cDNA was amplified according to the instructions of the PCR fluorescence quantitative kit of TransGen Biotech Company (Beijing, China). The primer sequences are listed in Table 1.

**Table 1:** **Primer sequences**

Gene name	Primer sequences
IPS-1	F:5'- GGGATTTGAGTGCTGCTCCT -3' R:5'- GTCTCCACCAACCTCACTGG -3'
MDA5	F:5'-TGGTCACATACAGCTCCAAGA-3' R:5'-ACGAGGAGATCAGCGTGTTG-3'
TRAF6	F:5’-TGGAAGGTCGTCTGGTGAGA-3’ R:5’-TGCCATGTGAGTGTTTTGCG-3’
TAK1	F:5’- TAATGACCCGCTGTTGGTCC-3’ R:5’- AGTACCGCATCAAGTGTGTCA-3’
IKKα	F:5'- CCTGCAAGTGTGGAAACACC-3' R:5'- ATGGAGAAAAGTGCCTGCGA-3'
IKKβ	F:5'- CCTGCAAGTGTGGAAACACC-3' R:5'- ATGGAGAAAAGTGCCTGCGA-3'
NF-κB P65	F:5'- AGGAGGGGTCTATGGAACAA-3' R:5'- TGTTGATGGAGGAGCTTCGG-3'
NF-κB P50	F:5’- CACGGAGGCTTGATCCTGTT-3’ R:5’- CCGCTGTCCTGTCCATTCTT-3’
NLRP3	F:5’- AGCCAAACCACTGGAAACCA-3’ R:5’- GGCAATGAGCACAGAGGACT-3’
Caspase-1	F:5’- GGCCAGGGAGATGTGCATAG-3’ R:5’- CTCCCGTGGCTGGTATATGTC-3’
IL-1β	F:5’- CATGTCGTGTGTGATGAGCG-3’ R:5’- TGTCGATGTCCCGCATGA-3’
IL-18	F:5’- AAGCGTGGCAGCTTTTGAAG-3’ R:5’- CTGAAGGTGCGGTGGTTTTG-3’
NIBV-N	F:5’-GGTAGYGGYGTTCCTGATAA-3’ R:5’-TCATCTTGTCRTCACCAAAA-3’
GAPDH	F:5’-TGGCATCCAAGGAGTGAGC-3’ R:5’-GGGAGACAGAAGGGAACAG-3’

### Viral strains and viral load determination

The NIBV virulent strains used in this experiment were isolated and preserved in the Clinical Veterinary Laboratory, College of Animal Science and Technology, Jiangxi Agricultural University (SX9, accession number: MN707951.1). The pMD18-T-N-positive plasmid constructed in our laboratory was used as a standard and was diluted to 8 concentrations (10^3^, 10^4^, 10^5^, 10^6^, 10^7^, and 10^8^), and the Ct values were obtained by RT‒PCR. The logarithmic value of the plasmid concentration was used as the abscissa, and the Ct value obtained during the reaction was used as the ordinate to construct the standard curve. Then, the cell cDNA was amplified via RT‒PCR, and the copy number of the virus was calculated by the standard curve. The upstream and downstream primers used were N gene primers from our laboratory.

### Western blotting

Total protein was extracted from the samples with RIPA lysis buffer (Beyotime Institute of Biotechnology, Jiangsu, China). The concentration of the protein lysate was measured by a spectrophotometer, and the protein was subsequently diluted to a uniform concentration. Total protein with loading buffer was heated to 100 °C for 10 min and then run on 12% SDS‒PAGE gels. The separated proteins were transferred to PVDF Western blotting membranes (Merck Millipore, IPVH00010), after which all the membranes were treated with PBS supplemented with 5% skim milk powder at 4 °C for 30 min and then incubated with the following primary antibodies: anti-NLRP3, anti-procaspase1, anti-caspase1 p20, anti-IL-18, anti-IL-1β, anti-IPS-1, anti-TRAF6, anti-NF-κB p65, anti-NF-κB p50, and anti-NIBV-N. GAPDH was used as the loading control. Next, the membranes were incubated with a peroxidase-conjugated secondary antibody. After the membranes were incubated with primary and secondary antibodies, a Bio-Rad gel imager was used to photograph the protein bands, and ImageJ was used to analyse the protein bands.

### Immunofluorescence

After a small drop of PBS was added to the cell culture plate, the sterilized cell crawl sheet was placed, and primary chicken renal tubular epithelial cells were extracted and inoculated on the cell crawl sheet at a density of 1 × 10^5^ cells/mL. The medium was discarded when the cells grew to the appropriate density, 1 mL of paraformaldehyde was added to each well, and the cells were fixed at room temperature for 2 h and then placed in a refrigerator at 4 °C. After staining, the return film was obtained, and NLRP3 fluorescence was photographed in a laser confocal chamber.

### Statistics

All the data are presented as the means ± standard errors of the means (SEMs). The statistical significance of the mean values in two-group comparisons was determined using Student’s *t* test. The data from each group were independent and showed equal variances (Levene’s test) and a normal distribution. One-way analysis of variance (ANOVA followed by Tukey’s test for more than two groups) was applied to determine statistical significance. *P* < 0.05, *P* < 0.01 and *P* < 0.001 are indicated by *, ** and ***, respectively. The data map was plotted with GraphPad Prism 10.0.0 and showed significance.

## Results

### NIBV leads to injury and death of primary renal tubular epithelial cells

**Figure 1:**
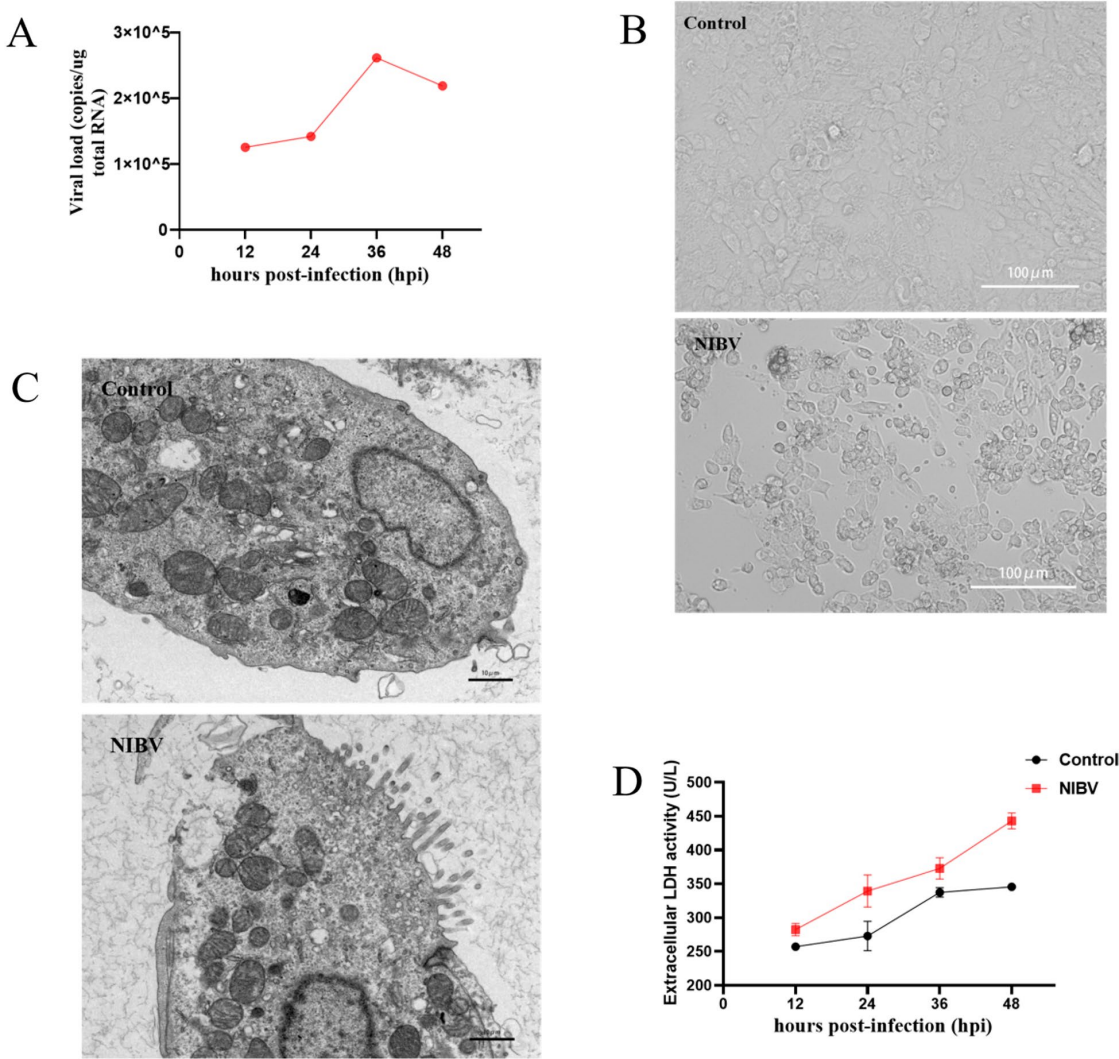
**NIBV infection induced renal tubular epithelial cell injury and death. A** Copy numbers of NIBV-infected cells detected by RT–PCR: 12, 24, 36, 48 hpi (*n* = 4). **B** Microscopically normal primary renal tubular epithelial cells and NIBV-infected primary renal tubular epithelial cells. Scale bar: 100 μm. **C** Electron microscopy of normal primary renal tubular epithelial cells and NIBV-infected primary renal tubular epithelial cells. Scale bar: 10 μm. **D** LDH levels in the cell supernatant detected at 12, 24, 36 and 48 hpi after NIBV infection in U/L (*n* = 3).

NIBV can infect renal tubular epithelial cells, resulting in nephritis, necrosis, and renal tubular nephropathy in chicks [14]. We detected the viral load of primary chicken renal tubular epithelial cells after NIBV infection and reported that the copy number of NIBV in the cells increased with time from 12 h post-infection (hpi) to 36 hpi (Figure 1A). However, there was a decreasing trend at 48 hpi, which may have occurred because NIBV led to cell shedding and death; thus, the amount of viral RNA in the collected total cell RNA decreased. Next, we observed the cells at the peak of viral infection, i.e., 36 hpi. Under the microscope, compared with control cells, NIBV-infected cells exhibited vacuolation and shedding, and the cells became round and wrinkled and formed clusters (Figure 1B). High-power microscopy (High-Tech, Japan) revealed that the cells in the NIBV group exhibited lesions characteristic of pyroptosis, that is, cell membrane rupture (Figure 1C). In addition, the LDH activity in the supernatant increased in a manner dependent on the duration of virus infection (Figure 1D); compared with that in the control group, the LDH activity in the supernatant was greater in the NIBV group.

### NIBV induced pyroptosis in primary chicken renal tubular epithelial cells

**Figure 2:**
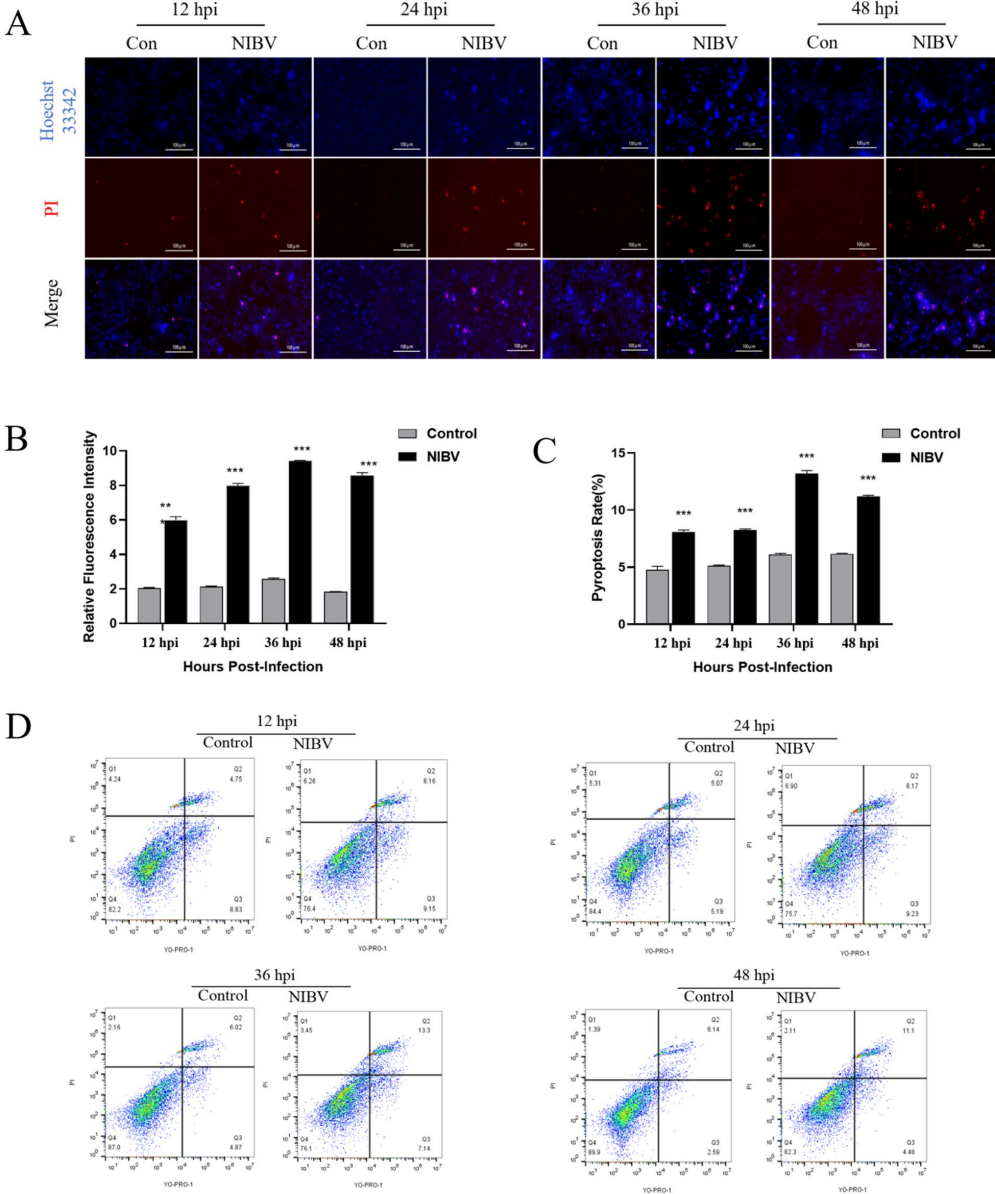
**NIBV infection induced pyroptosis in primary chicken renal tubular epithelial cells. A** Hoechst/PI staining of cells at four time points; blue, Hoechst; red, PI. Scale bar: 100 μm (*n* = 3). **B** Quantification of Hoechst/PI staining. For statistical analysis, one-way ANOVA and the Bonferroni post hoc correction were performed. All values are expressed as average values; *P < 0.05, **P < 0.01, and ***P < 0.001. **C**, **D** Detection of YO-PRO-1 and PI double-positive cells at four time points by flow cytometry and a positive inducer of pyroptosis (*n* = 3).

**Figure 3:**
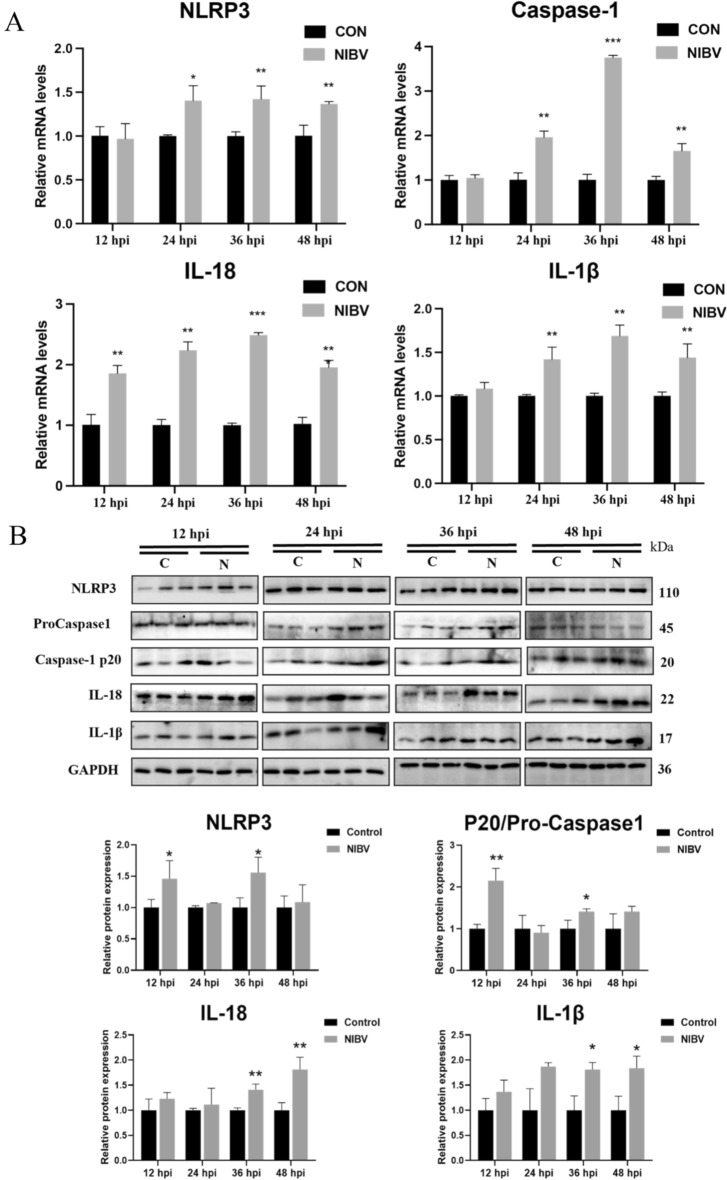
**NIBV infection activates the classical pyroptosis signalling pathway. A** RT‒PCR analyses of genes related to the classical signalling pathway of pyroptosis at 12, 24, 36, and 48 hpi (NLRP3, caspase-1, IL-18, and IL-1), with GAPDH used as the normalization control (*n* = 4). **B** WB analyses of proteins related to the classical signalling pathway of pyroptosis (NLRP3, pro-caspase-1, caspase-1 p20, IL-18 and IL-1β). Densitometry quantifications are presented below using ImageJ (*n* = 3). GAPDH was utilized as a loading control. The data are normalized as a control group. For statistical analysis, one-way ANOVA and the Bonferroni post hoc correction were performed. All values are expressed as average values; *P < 0.05, **P < 0.01, and ***P < 0.001.

Compared with those in the control group, the number of Hoechst-PI double-positive cells in the NIBV group was significantly greater, and the difference was most obvious at 36 hpi (Figure 2A). Compared with that in the control group, the proportion of YO-PRO-1/PI double-positive cells in the NIBV group was also significantly greater, and the proportion of YO-PRO-1/PI double-positive cells was greatest at 36 hpi (Figure 2D). These findings suggest that NIBV infection causes pyroptosis in primary tubular epithelial cells. To further explore the molecular mechanism of pyroptosis induced by NIBV, the levels of genes and proteins related to the classical signalling pathway of pyroptosis were detected (Figures 3A, B). After NIBV infection, the mRNA expression levels of genes related to the NLRP3 classic pathway (NLRP3, caspase-1, IL-18 and IL-1β) significantly increased at 24, 36, and 48 hpi, and the protein expression levels of IL-18 and IL-1β significantly increased at 36 and 48 hpi. The results indicated that the assembly of the NLRP3 inflammasome induced by NIBV infection facilitated the activation of caspase-1, thus promoting the release of IL-18 and IL-1β, suggesting that NIBV triggered the NLRP3 classical pyroptosis pathway in primary chicken renal tubular epithelial cells.

### NIBV activated the MDA5/NF-κB signalling pathway in primary chicken renal tubular epithelial cells

**Figure 4:**
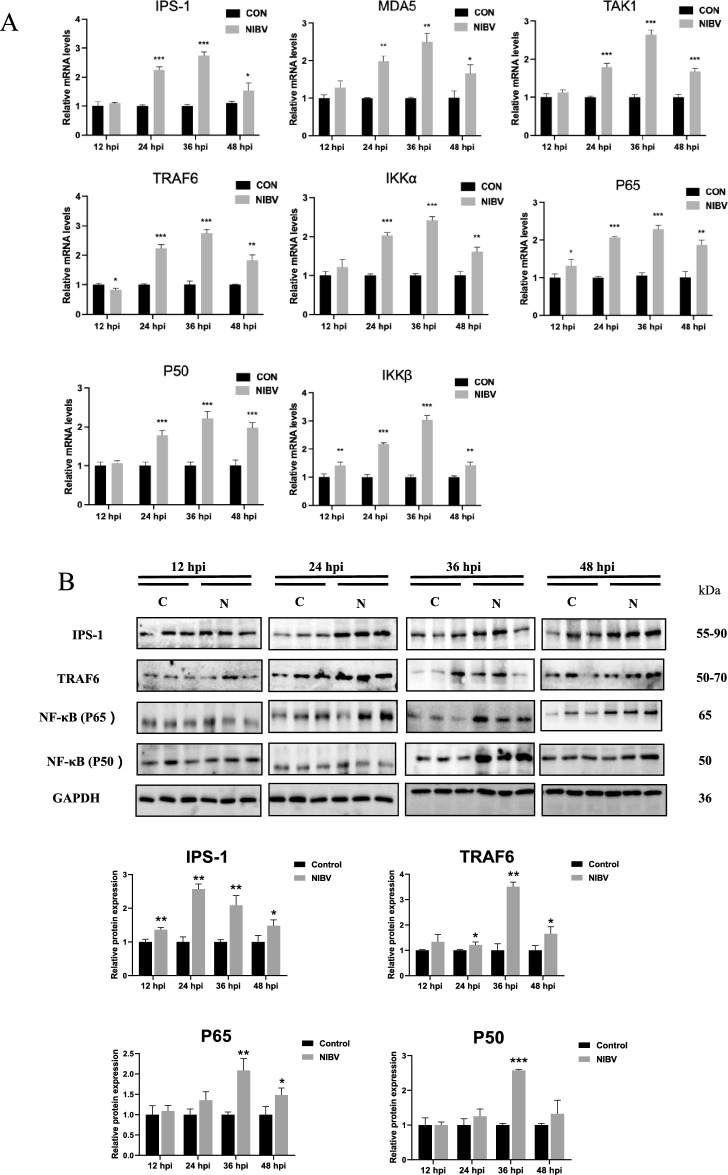
**MDA5 recognizes NIBV and activates MDA5/NF-κB immune signalling pathway transduction. A** RT‒PCR analysis of genes related to the MDA5/NF-κB immune signalling pathway (MDA-5, IPS-1, TRAF6, TAK1, IKKα, IKKβ, P65 and P50). GAPDH was used as the normalization control (*n* = 4). **B** WB analyses of proteins related to the MDA5/NF-κB immune signalling pathway (IPS-1, TRAF6, NF-κB (P65), and NF-κB (P50)) (*n* = 3). Densitometry quantifications are presented below using ImageJ. GAPDH was used as a loading control. The data are normalized as a control group. For statistical analysis, one-way ANOVA and the Bonferroni post hoc correction were performed. All values are expressed as average values; *P < 0.05, **P < 0.01, and ***P < 0.001.

Studies have shown that MDA5 signalling activates IPS-1 signalling, which can trigger the activation of downstream signals, cause the release of antiviral factors such as interferon, and induce innate immune responses to control viral infection [8]. However, at the peak of NIBV infection, the overactivation of MDA5 receptors leads to the dysregulation of various complex signals. Here, to determine whether NIBV can be recognized by MDA5 and upregulate the activity of the MDA5/NF-κB signalling pathway, the expression of MDA5/NF-κB signalling pathway-related factors was detected. Compared with those in the control group, the mRNA expression levels of genes related to this signalling pathway (MDA5, IPS-1, TRAF6, TAK1, IKKα, IKKβ, P65, and P50) significantly increased at 24, 36, and 48 hpi after NIBV infection (Figure 4A), and the expression levels of related proteins significantly increased at 36 hpi (Figure 4B). These findings suggest that NIBV can activate the MDA5/NF-κB immune signalling pathway.

### Inhibition of TRAF6 reduces NIBV replication and alleviates cell damage

**Figure 5:**
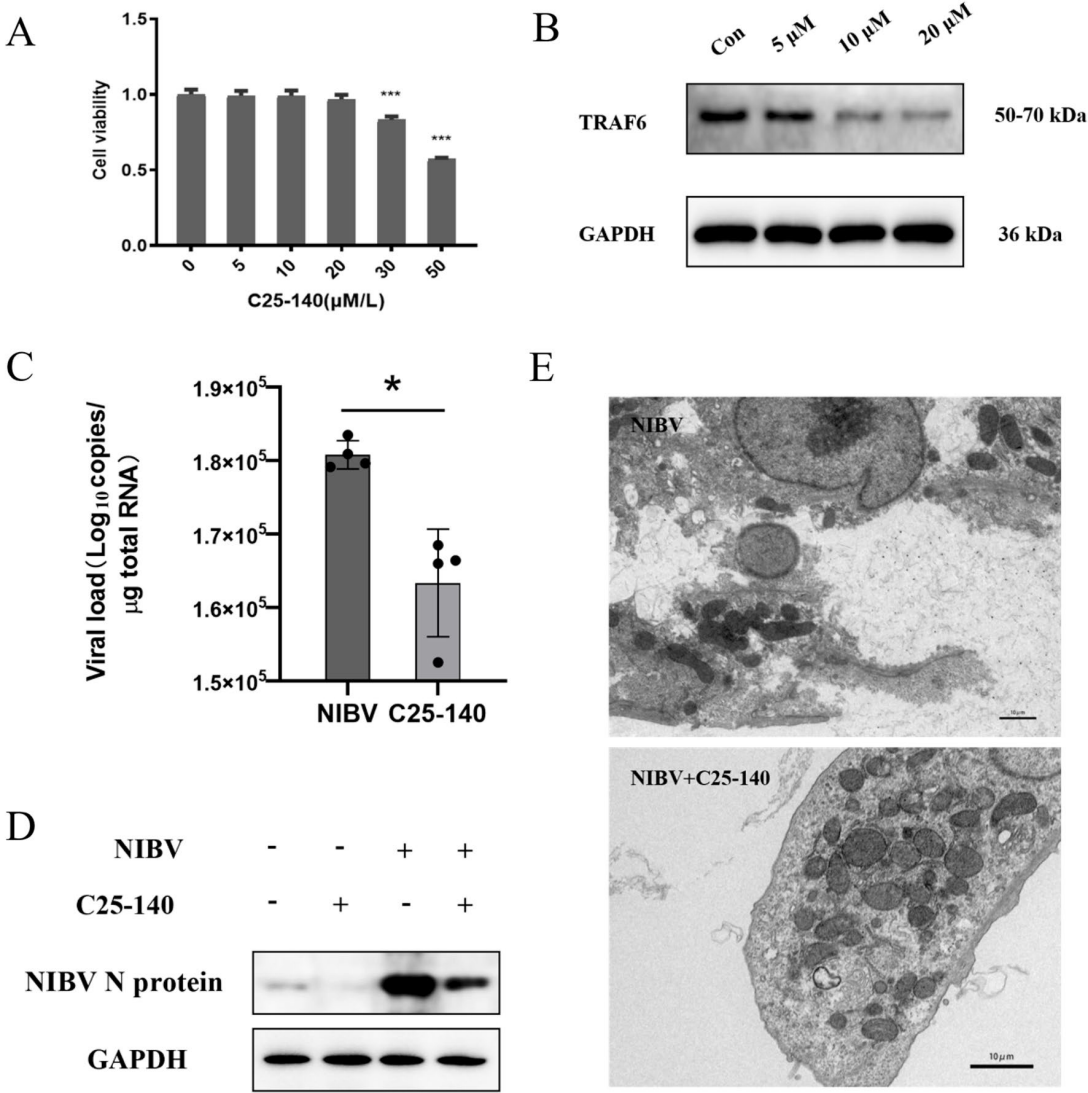
**C25-140, which targets TRAF6, can alleviate the damage to primary chicken renal tubular epithelial cells treated with NIBV and reduce the degree of NIBV replication. A** Cell viability of chicken renal tubular epithelial cells treated with different concentrations of the inhibitor for 36 h. **B** TRAF6 protein expression in chicken renal tubular epithelial cells treated with different concentrations of TRAF6 for 36 h. **C** After the cells were treated with 20 μM C25-140 and 1 MOI NIBV for 2 h and then cultured in normal medium for 36 h, the total RNA concentration of the cells was quantitatively detected by real-time fluorescence quantitative PCR (*n* = 4). **D** The content of NIBV N protein in the extracted total cell protein was detected using an anti-NIBV N protein antibody. **E** Primary tubular epithelial cells infected with NIBV and treated with C25-140 were observed by transmission electron microscopy. Scale bar: 10 μm. *P < 0.05, **P < 0.01, ***P < 0.001.

TRAF6 plays an important pivotal role upstream of the MDA5/NF-κB immune signalling pathway and is a necessary junction molecule for this signalling pathway [15]. To elucidate the relationship between the activity of the MDA5/NF-κB signalling pathway and pyroptosis, we evaluated the cytotoxicity of different concentrations of TRAF6 inhibitors in primary renal tubular epithelium by a CCK8 assay (Figure 5A). The inhibitory effect of different concentrations of C25-140 on TRAF6 cells was verified by WB. We found that 20 μM is the maximum nontoxic concentration for cells (Figure 5B); thus, we used 20 μM C25-140 for subsequent experiments. Coincubation of C25-140 with NIBV reduced the replication of NIBV in cells (Figures 5C, D), and transmission electron microscopy revealed that C25-140 had a protective effect on NIBV-infected cell morphology (Figure 5E).

### The absence of TRAF6 in primary chicken renal tubular epithelial cells mitigated NIBV-induced pyroptosis

**Figure 6:**
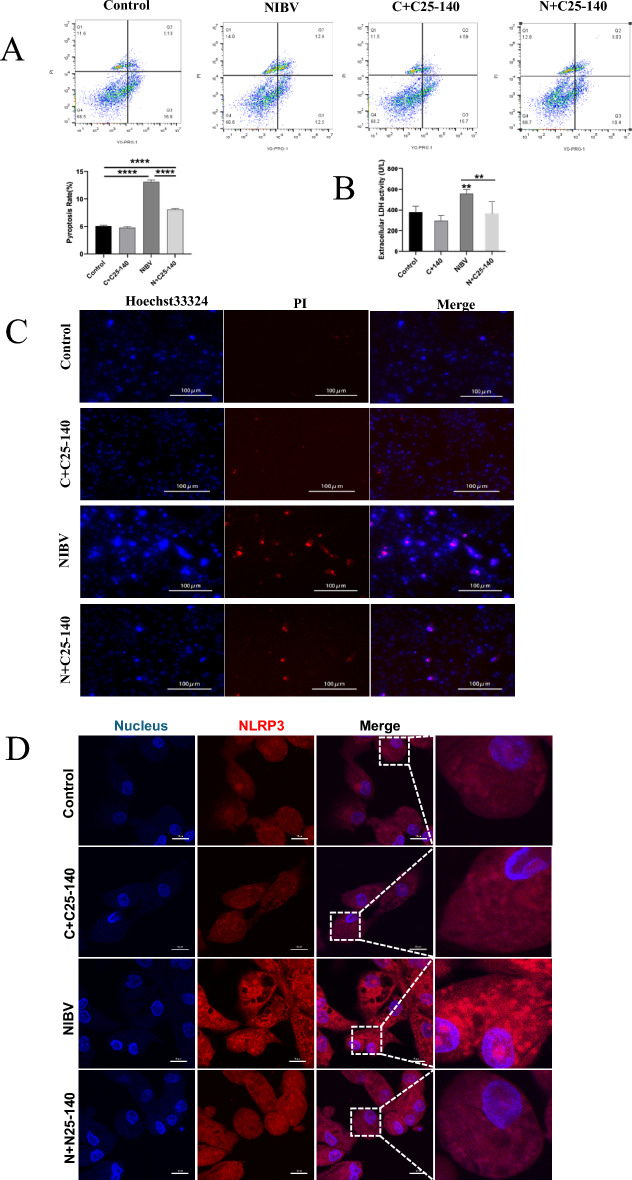
**The inhibition of TRAF6 can reverse NIBV-induced pyroptosis in primary chicken renal tubular epithelial cells. A** Cells were collected after the administration of 20 μM TRAF6 inhibitor. A YO-PRO-1/PI apoptosis and necrosis detection kit and flow cytometry were used to identify cells from the four time periods. **B** The flow cytometry results are shown as a quantification chart of YO-PRO-1 and PI comarkers, with all the values expressed as averages; *P < 0.05, ** *P* < 0.01, and *** *P* < 0.001. **C** Hoechst-PI staining of cells; blue, Hoechst; red, PI. Scale bar: 100 μm; *n* = 3. **D** NLRP3 protein staining was observed under a confocal laser microscope (blue, DAPI; red, NLRP3; Scale bar: 10 μm; *n* = 3).

Primary chicken renal tubular epithelial cells were divided into a control group, a control + C25-140 group, a NIBV group and a NIBV + C25-140 group for culture, after which immunofluorescence staining and flow cytometry were performed. YO-PRO-1/PI flow cytometry and Hoechst-PI staining revealed that the proportion of TRAF6-deficient cells with NIBV-induced pyroptosis was reduced (Figures 6A–C). The inhibition of REAF6 expression reduced the number of NLRP3-positive events after NIBV infection (Figure 6D). In conclusion, the absence of TRAF6 in primary chicken renal tubular epithelial cells inhibited NIBV-induced pyroptosis.

### NIBV induces pyroptosis via the MDA5/NF-κB/NLRP3 signalling pathway

**Figure 7:**
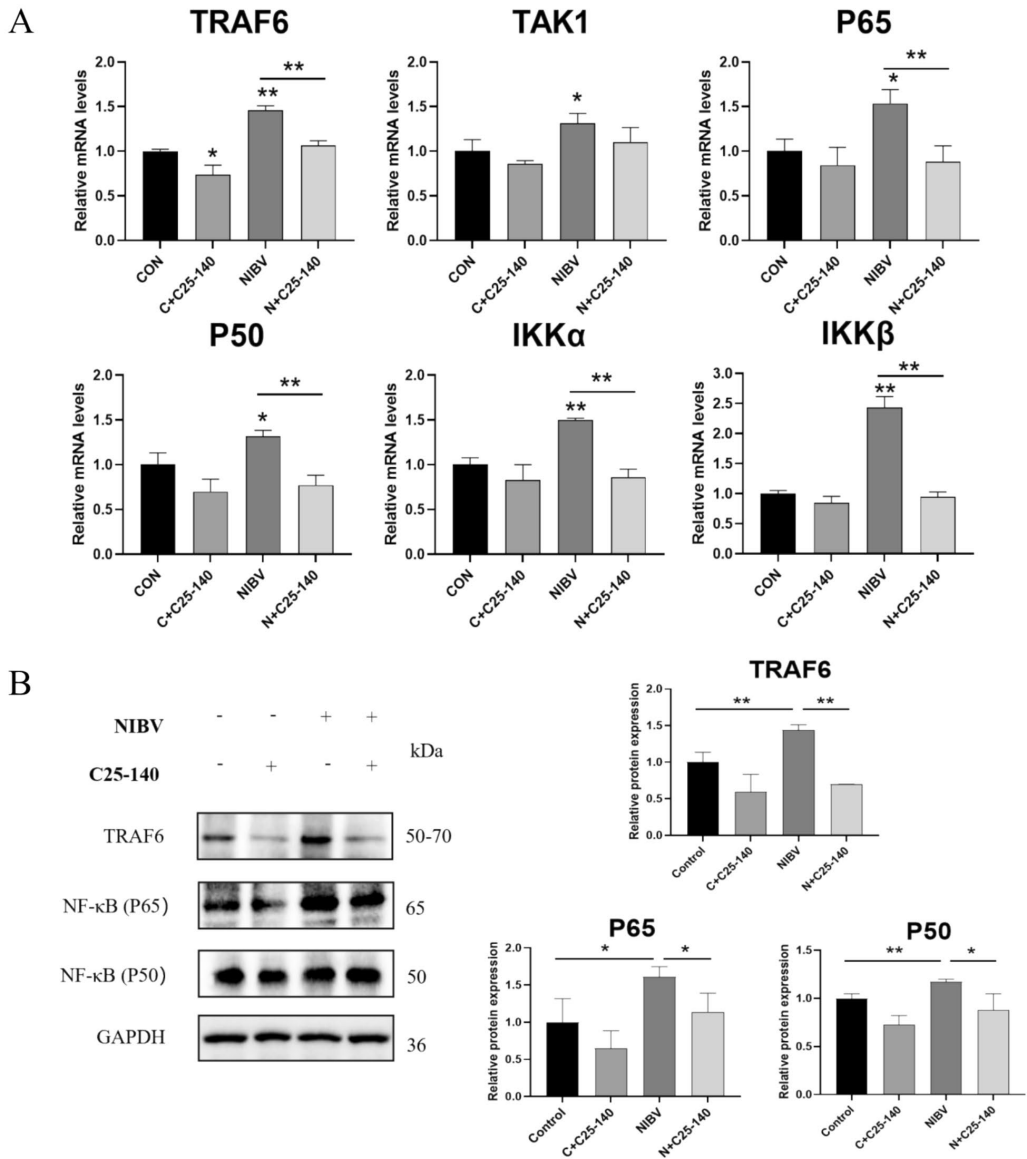
**The inhibition of TRAF6 activity partially blocks the activity of the MDA5/NF-κB immune signalling pathway. A** Total RNA and proteins were extracted from cells treated with a TRAF6 inhibitor at a concentration of 20 μM. RT‒PCR analysis of genes related to the MDA5/NF-κB immune signalling pathway (TRAF6, TAK1, P65, P50, IKKα, and IKKβ); GAPDH was used as the normalization control. (*n* = 4). **B** WB analyses of proteins related to the MDA5/NF-κB immune signalling pathway. (*n* = 3). Densitometry quantifications are presented below using ImageJ. GAPDH was used as a housekeeping gene to normalize the results. The data are normalized as a control group. For statistical analysis, one-way ANOVA and the Bonferroni post hoc correction were performed. All values are expressed as average values; * *P* < 0.05, ** *P* < 0.01, and *** *P* < 0.001.

**Figure 8:**
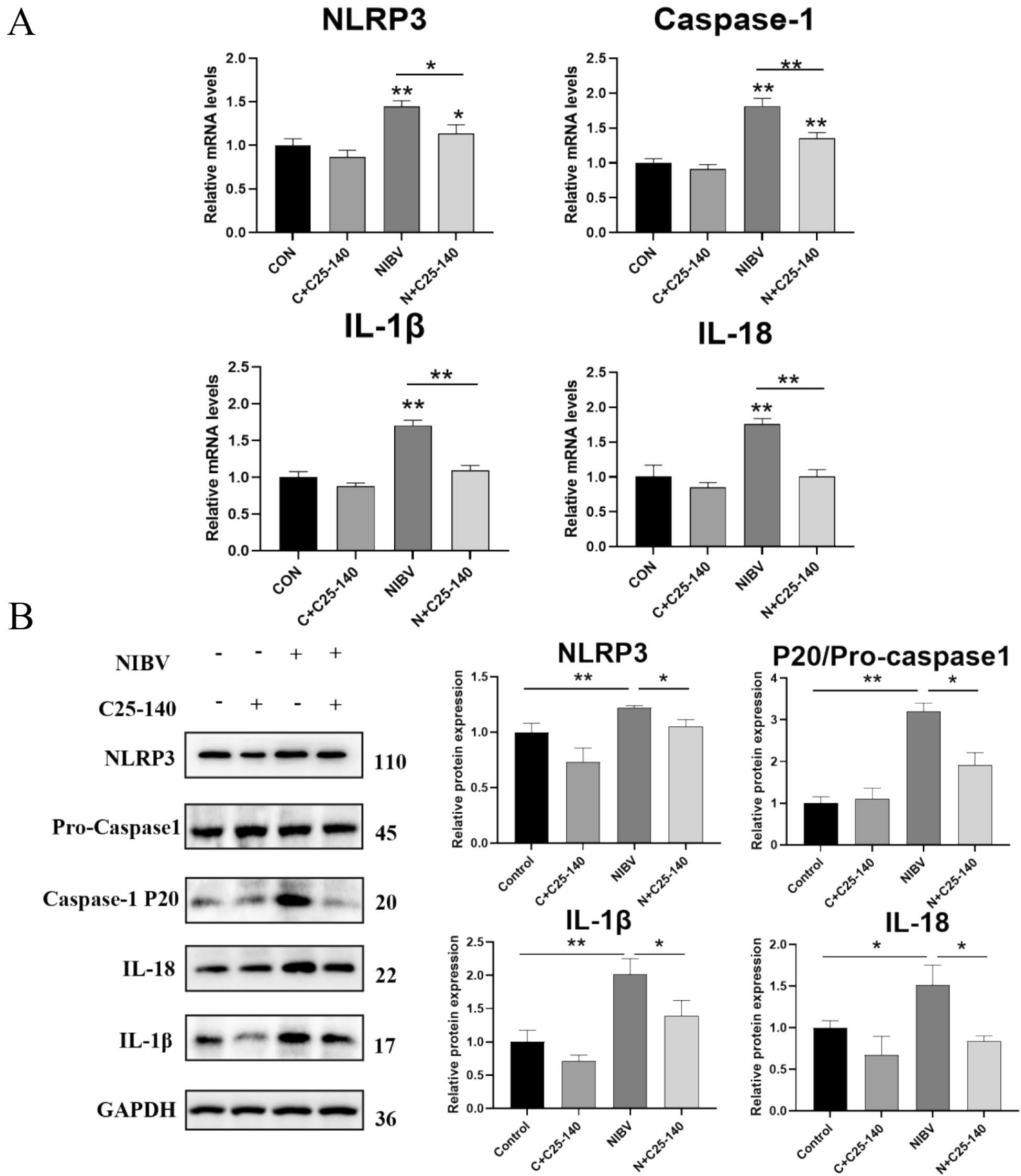
**C25-140, which targeted TRAF6, reversed the activation of the classical pyroptosis signalling pathway by NIBV.** RNA and the protein in the cells extracted at 36 hpi. **A** RT‒PCR analysis of genes related to the pyroptosis NLRP3 classical pathway; GAPDH was used as the normalization control. (*n* = 4). **B** WB analyses of proteins related to the classical pyroptosis NLRP3 pathway. (*n* = 3). Densitometry quantifications are presented below using ImageJ. GAPDH was used as a loading control. The data are normalized as a control group. For statistical analysis, one-way ANOVA and the Bonferroni post hoc correction were performed. All values are expressed as average values; * *P* < 0.05, ** *P* < 0.01, and *** *P* < 0.001.

Compared with those in the NIBV group, gene and protein expression associated with the MDA5/NF-κB signalling pathway (TRAF6, TAK1, P65, P50, IKKα and IKKβ) were significantly downregulated in the NIBV + C25-140 group (Figures 7A, B). Compared with those in the NIBV group, the genes and protein expression associated with the classical NLRP3 pathway of pyroptosis were significantly downregulated in the NIBV + C25-140 group (Figures 8A, B). These findings suggest that C25-140 inhibits the classical NLRP3 pyroptosis pathway, which is activated by NIBV. These results indicate that the MDA5/NF-κB/NLRP3 signalling pathway plays a key role in the antipyroptotic effect of C25-140 on NIBV.

## Discussion

As a programmed cell death mechanism, pyroptosis is closely related to the inflammatory response, and NIBV infection causes a severe inflammatory response in the kidney. MDA5 recognizes RNA viruses and promotes the transcription of inflammatory factors, activating the transcription factor NF-κB, which plays a key role in the activation of the NLRP3 inflammasome. We hypothesized that NIBV induces chicken nephritis by activating pyroptosis via the MDA5/NF-κB/NLRP3 signalling pathway. Our study established a model of NIBV infection in chicken renal tubular epithelial cells and revealed that NIBV activated the MDA5/NF-κB/NLRP3 signalling pathway and upregulated the expression of markers associated with pyroptosis. Activation of this pathway was reversed by treatment with the key gene of the TRAF6 (MDA5/NF-κB pathway) inhibitor C25-140, and the proportion of tubular epithelial cells with pyroptosis markers was reduced. These findings demonstrated that NIBV induces inflammatory responses in chicken kidneys by activating pyroptosis via the MDA5/NF-κB/NLRP3 signalling pathway.

NIBV can cause kidney inflammation and urate deposition, which can lead to kidney failure and necrosis in severe cases. Studies have shown that the damage caused by NIBV to chicken kidneys is closely related to the viral load in the tissue [16]. In accordance with the results of previous studies in our laboratory, we screened suitable concentrations of NIBV through the determination of TCID50, among which the 4th passage virus prepared in the laboratory was the most effective and had moderate toxicity to cells [17]. According to the experimental results shown in Figure 1A, the culture time of NIBV-infected cells was determined to be 36 h. During the culture process, the cells were deformed and rounded, with large vacuoles in the cytoplasm and large shedding in severe cases, which was consistent with the expectation that NIBV could cause damage to and pyroptosis of primary renal tubular epithelial cells. These findings demonstrate that pyroptosis is essential for the cellular damage caused by NIBV infection of chicken renal tubular epithelial cells [18]. However, overactivation of pyroptosis can lead to a violent inflammatory response and cell death [19]. These studies have shown that in the NIBV chick model, the pyroptosis signalling pathway is activated, enabling the NLRP3 inflammasome to activate caspase-1 and induce inflammation. On this basis, the molecular mechanism was further elucidated in this study.

Our results revealed that LDH activity in the cell supernatant increased in a time-dependent manner after the infection of chicken renal tubular epithelial cells, indicating that NIBV could damage the cell membrane of chicken renal tubular epithelial cells and release a large amount of lactate dehydrogenase in a time-dependent manner. In this study, NIBV increased the pyroptosis rate of chicken renal tubular epithelial cells, reaching 13.3% at 36 hpi. According to the Hoechst/PI staining results, the degree of pyroptosis in chicken renal tubular epithelial cells also increased significantly at 36 hpi. In addition, NIBV infection increased the gene and protein levels of NLRP3, caspase-1, IL-1β and IL-18 and peaked especially at 36 hpi. The main purpose of viral infection is to carry out viral replication and produce and spread new virions. It has been reported that cell death caused by stress induced by viral replication contributes to the release of viral progeny at the end of infection [1], while pyroptosis, an important form of programmed cell death, plays a role mainly through the classical pyroptosis pathway. First, the NLRP3 inflammasomes activate caspase-1 [20]. After caspase is activated, Pro-IL-1β and Pro-IL-18 are split into IL-1β and IL-18, respectively, and then released, after which N-GSDMD penetrates the cell membrane to a diameter of approximately 1–2 μm. Thus, mature IL-4β/IL-5 and caspase-5 with a diameter of 1.18 nm can pass through [21]. Owing to the formation of pores, liquids such as tissue fluid or medium enter the cytoplasm, causing the cells to expand, rupture, permeate and dissolve, thus releasing a large number of inflammatory factors, such as IL-1β and IL-18 [22]. Our experimental results are consistent with the significant upregulation of pyroptosis-related genes and proteins after NIBV infection, and these results suggest that NIBV causes pyroptosis in renal tubular epithelial cells.

Melanoma differentiation-associated gene 5 (MDA5) belongs to the RIG-I-like receptors (RLRs) of pattern recognition receptors (PRRs), whose main functions include recognizing intracellular viral RNA and triggering the innate immune response. Viral ligands are molecules on the surface of a virus that bind to specific receptor proteins on a host cell, enabling the virus to infect that cell. MDA5 detects RNA viral ligands or transcriptional intermediates in the cytoplasm and is subsequently able to stimulate innate immunity and inflammation and trigger a cascade of activation signals. Studies have shown that MDA5 can cooperate with other RLRs, such as Toll receptors, to trigger a weaker signalling network, indicating that it can be directly activated by viral signals [23]. Previous studies have shown that NIBV can mediate kidney injury by activating the TLR7/NF-κB signalling axis in the kidney [24] and that the virus can induce apoptosis signalling, connecting the GRP78/PERK/ATF-4 signalling pathway in tandem [16].

We hypothesized that NIBV activates the MDA5/NF-κB innate immune signalling pathway. Therefore, after conducting experiments related to cell pyroptosis, we further explored the effect of NIBV on the innate immune signalling pathway of MDA5/NF-κB and found that the expression of genes related to this signalling pathway (MDA5, IPS-1, TRAF6, TAK1, P65, P50, IKKα and IKKβ) was upregulated. In addition, TRAF6, P65 and IKKβ are significantly activated at the early stage of NIBV infection, and NIBV can significantly increase the protein levels of IPS-1, TRAF6, P65 and P50, and most of the proteins are most significantly expressed at 36 hpi after infection with the virus. Notably, IPS-1 protein expression was significantly upregulated at 12 hpi, possibly because MDA5 was able to recognize dsRNA, the replication intermediate of NIBV in the cytoplasm, and the recognition of RNA by MDA5 caused a cascade reaction of mitochondrial protein IPS-1 [25, 26]. These findings confirm our hypothesis that nephropathogenic infectious bronchitis activates the MDA5/NF-κB innate immune signalling pathway [27].

RIG-I-like receptors play important roles in the host’s innate immunity against viruses, in which MDA5 is involved in the host’s cell death mechanism. Following viral infection and FasL treatment, IPS-1-mediated proteolytic cleavage and the isolation of the N-terminal CARD domain from the MDA-5 helicase domain have been shown to accelerate apoptosis [28]. MDA5 is thought to be involved in IL-1β mRNA transcriptional activation and LDH release in respiratory virus immune pyroptosis [29]. These studies also explain the activation of MDA5 in the process of NIBV-induced pyroptosis.

However, the exact molecular mechanism underlying the relationship between MDA5 and pyroptosis remains unclear. To further explore the relationship between this signalling pathway and pyroptosis, we hypothesized that MDA5 signalling pathway transduction is related to NIBV-induced pyroptosis. We subsequently inhibited the expression of TRAF6, a key gene of the MDA5 signalling pathway. C25-140, a potent inhibitor of TRAF6, was selected, and it was found that the inhibitor could not only downregulate the activation of the MDA5/NF-κB innate immune signalling pathway by NIBV but also decrease the expression level of genes related to cell pyroptosis caused by NIBV, which was consistent with our hypothesis. One study revealed that pyroptosis induced by TRAF6 activated the NF-κB/NLRP3 axis [15]. This, in turn, further explains our hypothesis and results [13]. According to the results of NLRP3 immunofluorescence, the inhibitor can reverse the activation of NLRP3 by NIBV, and the flow cytometry results revealed that the pyroptosis rate of the cells treated with the inhibitor was significantly lower than that in the single challenge group. This indicated that the inhibitor could significantly inhibit the activation of pyroptosis by NIBV, which was also verified by the decrease in LDH activity. These results suggest that NIBV can regulate pyroptosis in chicken renal tubular epithelial cells through the MDA5/NF-κB innate immune signalling pathway. In addition, TRAF6 inhibitors reduced viral replication in chicken renal tubular epithelial cells, indicating that the inhibitor significantly inhibited nephropathogenic infectious bronchitis virus replication.

Our study suggests that NIBV induces the activation of the MDA5/NF-κB/NLRP3 signalling pathway, which mediates pyroptosis. The inhibition of TRAF6 mitigated NIBV-induced pyroptosis and reduced the viral replication of NIBV. The key role of the MDA5/NF-κB innate immune signalling pathway in NIBV-induced pyroptosis of primary renal tubular epithelial cells was demonstrated. The data that support the findings of this study are openly available in figshare. Reference number 10.6084/m9.figshare.30264160

## References

[1] Hoerr FJ (2021) The pathology of infectious bronchitis. Avian Dis 65:600–611 35068104 10.1637/aviandiseases-D-21-00096

[2] Xu P, Liu P, Zhou C, Shi Y, Wu Q, Yang Y, Li G, Hu G, Guo X (2019) A multi-omics study of chicken infected by nephropathogenic infectious bronchitis virus. Viruses 11:1070 31744152 10.3390/v11111070 PMC6893681

[3] Rao Z, Zhu Y, Yang P, Chen Z, Xia Y, Qiao C, Liu W, Deng H, Li J, Ning P, Wang Z (2022) Pyroptosis in inflammatory diseases and cancer. Theranostics 12:4310–4329 35673561 10.7150/thno.71086 PMC9169370

[4] Broz P, Ruby T, Belhocine K, Bouley DM, Kayagaki N, Dixit VM, Monack DM (2012) Caspase-11 increases susceptibility to *Salmonella* infection in the absence of caspase-1. Nature 490:288–291 22895188 10.1038/nature11419 PMC3470772

[5] Li Y, Qi Q, Chen Y, Ding M, Huang M, Huang C, Liu P, Gao X, Guo X, Zheng Z (2025) RIPK3 activation of CaMKII triggers mitochondrial apoptosis in NIBV-infected renal tubular epithelial cells. Vet Microbiol 302:110375 39808936 10.1016/j.vetmic.2025.110375

[6] Kato H, Takeuchi O, Sato S, Yoneyama M, Yamamoto M, Matsui K, Uematsu S, Jung A, Kawai T, Ishii KJ, Yamaguchi O, Otsu K, Tsujimura T, Koh CS, Reis e Sousa C, Matsuura Y, Fujita T, Akira S (2006) Differential roles of MDA5 and RIG-I helicases in the recognition of RNA viruses. Nature 441:101–105 16625202 10.1038/nature04734

[7] Li X, Wu K, Zeng S, Zhao F, Fan J, Li Z, Yi L, Ding H, Zhao M, Fan S, Chen J (2021) Viral infection modulates mitochondrial function. Int J Mol Sci 22:4260 33923929 10.3390/ijms22084260 PMC8073244

[8] Xu J, Jia Z, Chen A, Wang C (2020) Curcumin ameliorates *Staphylococcus aureus*-induced mastitis injury through attenuating TLR2-mediated NF-κB activation. Microb Pathog 142:104054 32061917 10.1016/j.micpath.2020.104054

[9] Goubau D, Deddouche S, Reise Sousa C (2013) Cytosolic sensing of viruses. Immunity 38:855–869 23706667 10.1016/j.immuni.2013.05.007 PMC7111113

[10] Lee AH, Shin HY, Park JH, Koo SY, Kim SM, Yang SH (2021) Fucoxanthin from microalgae *Phaeodactylum tricornutum* inhibits pro-inflammatory cytokines by regulating both NF-κB and NLRP3 inflammasome activation. Sci Rep 11:543 33436909 10.1038/s41598-020-80748-6 PMC7803995

[11] Li G, Fan Y, Lai Y, Han T, Li Z, Zhou P, Pan P, Wang W, Hu D, Liu X, Zhang Q, Wu J (2020) Coronavirus infections and immune responses. J Med Virol 92:424–432 31981224 10.1002/jmv.25685 PMC7166547

[12] Schneider CA, Rasband WS, Eliceiri KW (2012) NIH image to ImageJ: 25 years of image analysis. Nat Methods 9:671–675 22930834 10.1038/nmeth.2089 PMC5554542

[13] Ye J, Coulouris G, Zaretskaya I, Cutcutache I, Rozen S, Madden TL (2012) Primer-BLAST: a tool to design target-specific primers for polymerase chain reaction. BMC Bioinformatics 13:134 22708584 10.1186/1471-2105-13-134 PMC3412702

[14] Munuswamy P, Ramakrishnan S, Latheef SK, Kappala D, Mariappan AK, Kaore M, Anbazhagan K, Puvvala B, Singh KP, Dhama K (2021) First description of natural concomitant infection of avian nephritis virus and infectious bronchitis virus reveals exacerbated inflammatory response and renal damage in broiler chicks. Microb Pathog 154:104830 33691178 10.1016/j.micpath.2021.104830

[15] Sun D, Peng Y, Ge S, Fu Q (2022) USP1 inhibits NF-κB/NLRP3 induced pyroptosis through TRAF6 in osteoblastic MC3T3-E1 cells. J Musculoskelet Neuronal Interact 22:536–545 36458391 PMC9716302

[16] Chen W, Huang C, Shi Y, Li N, Wang E, Hu R, Li G, Yang F, Zhuang Y, Liu P, Hu G, Gao X, Guo X (2022) Investigation of the crosstalk between GRP78/PERK/ATF-4 signaling pathway and renal apoptosis induced by nephropathogenic infectious bronchitis virus infection. J Virol 96:e0142921 34669445 10.1128/JVI.01429-21 PMC8791289

[17] Ju X, Ding Q (2019) Hepatitis e virus assembly and release. Viruses 11:539 31181848 10.3390/v11060539 PMC6631228

[18] Jorgensen I, Zhang Y, Krantz BA, Miao EA (2016) Pyroptosis triggers pore-induced intracellular traps (PITs) that capture bacteria and lead to their clearance by efferocytosis. J Exp Med 213:2113–2128 27573815 10.1084/jem.20151613 PMC5030797

[19] Qi X (2016) Formation of membrane pores by gasdermin-N causes pyroptosis. Sci China Life Sci 59:1071–1073 27460194 10.1007/s11427-016-5109-3

[20] Ding J, Wang K, Liu W, She Y, Sun Q, Shi J, Sun H, Wang DC, Shao F (2016) Pore-forming activity and structural autoinhibition of the gasdermin family. Nature 535:111–116 27281216 10.1038/nature18590

[21] Vasudevan SO, Behl B, Rathinam VA (2023) Pyroptosis-induced inflammation and tissue damage. Semin Immunol 69:101781 37352727 10.1016/j.smim.2023.101781 PMC10598759

[22] Kudr J, Nguyen HV, Gumulec J, Nejdl L, Blazkova I, Ruttkay-Nedecky B, Hynek D, Kynicky J, Adam V, Kizek R (2014) Simultaneous automatic electrochemical detection of zinc, cadmium, copper and lead ions in environmental samples using a thin-film mercury electrode and an artificial neural network. Sensors (Basel) 15:592–610 25558996 10.3390/s150100592 PMC4327037

[23] Kang DC, Gopalkrishnan RV, Lin L, Randolph A, Valerie K, Pestka S, Fisher PB (2004) Expression analysis and genomic characterization of human melanoma differentiation associated gene-5, mda-5: a novel type I interferon-responsive apoptosis-inducing gene. Oncogene 23:1789–1800 14676839 10.1038/sj.onc.1207300

[24] Yu X, Wang H, Li X, Guo C, Yuan F, Fisher PB, Wang XY (2016) Activation of the MDA-5-IPS-1 viral sensing pathway induces cancer cell death and type i IFN-dependent antitumor immunity. Cancer Res 76:2166–2176 26893477 10.1158/0008-5472.CAN-15-2142 PMC4873369

[25] Alcock BP, Raphenya AR, Lau TTY, Tsang KK, Bouchard M, Edalatmand A, Huynh W, Nguyen AV, Cheng AA, Liu S, Min SY, Miroshnichenko A, Tran HK, Werfalli RE, Nasir JA, Oloni M, Speicher DJ, Florescu A, Singh B, Faltyn M, McArthur AG (2020) CARD 2020: antibiotic resistome surveillance with the comprehensive antibiotic resistance database. Nucleic Acids Res 48:D517–D525 31665441 10.1093/nar/gkz935 PMC7145624

[26] Kasumba DM, Hajake T, Oh SW, Kotenko SV, Kato H, Fujita T (2018) A plant-derived nucleic acid reconciles type I IFN and a pyroptotic-like event in immunity against respiratory viruses. J Immunol 200:1527 29330319 10.4049/jimmunol.1701732

[27] Yu L, Zhang X, Wu T, Su J, Wang Y, Wang Y, Ruan B, Niu X, Wu Y (2017) Avian infectious bronchitis virus disrupts the melanoma differentiation associated gene 5 (MDA5) signaling pathway by cleavage of the adaptor protein MAVS. BMC Vet Res 13:332 29132350 10.1186/s12917-017-1253-7 PMC5683607

[28] Li M-H, Si S-T, Y K, C Yu, Z Q, Y P (2018) Study of the molecular mechanism by which MDA5 mediates ACF cell pyroptosis induced by poly I:C. J Pathog Biol 5:1091–1095

[29] Liu W, Liu P, Wang T, Lin H, Huang Q, Deng G, Gao X, Guo X, Zhang C (2018) Changes of antioxidant function and the mRNA expression levels of xanthine oxidase in primary chick kidney cell culture caused by nephropathogenic infectious bronchitis virus infection. Pak Vet J 38:1

